# Supplementary material for: Gestational age and the risk of autism spectrum disorder in Sweden, Finland, and Norway: A cohort study
Source: PLoS Med. 2020 Sep 22;17(9):e1003207. doi: 10.1371/journal.pmed.1003207 (PMC7508401; doi:10.1371/journal.pmed.1003207)
Supplement: S2 Fig — ASD, autism spectrum disorder. (DOCX) [file pmed.1003207.s002.docx]

**Figure S2** Relative risks of Autistic Disorder for each week of gestational age compared to week 40. By country.


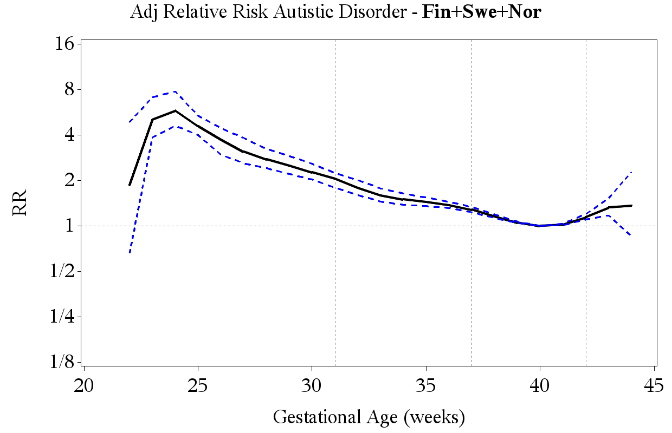

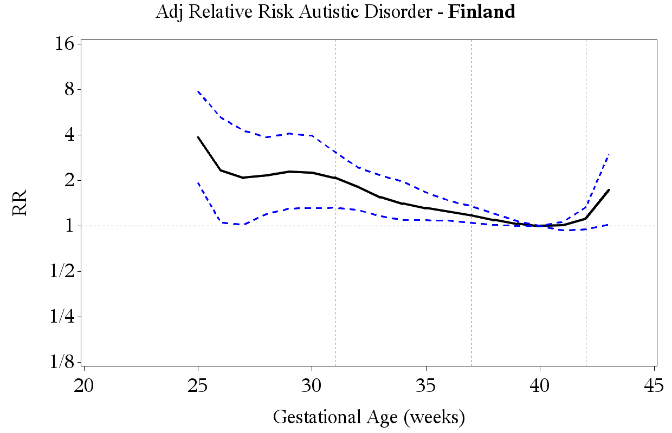


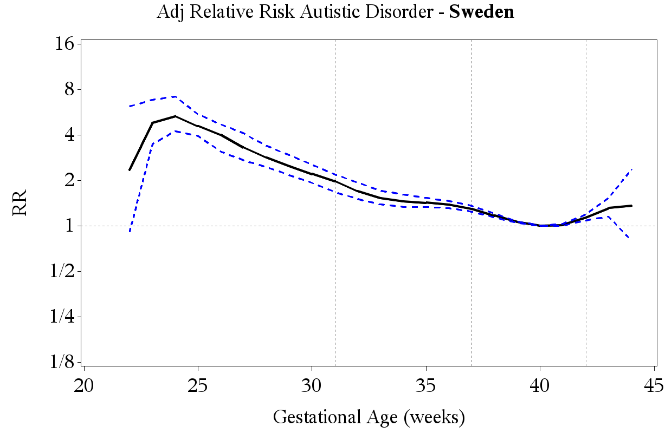

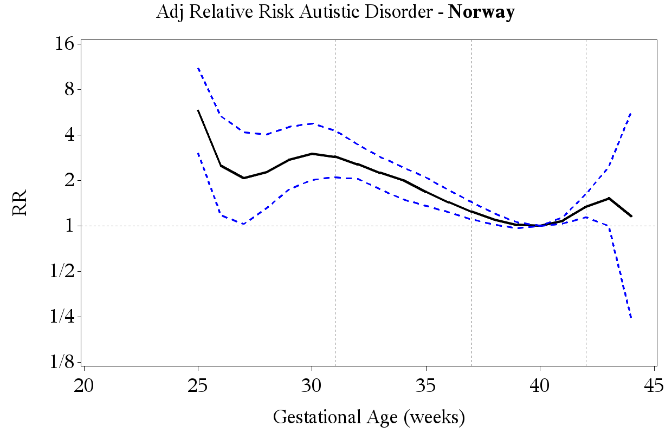


Relative risks (RR) of Autistic Disorder estimated by log-binomial regression adjusted for country (Finland, Sweden, Norway), Birth year (1995-1999, 2000-2004, 2005-2009, and 2010-20) and Maternal age (<20, 20-24, 25-29, 30-34, 35-39, and ≥40 years)
