# Supplementary material for: Gestational age and the risk of autism spectrum disorder in Sweden, Finland, and Norway: A cohort study
Source: PLoS Med. 2020 Sep 22;17(9):e1003207. doi: 10.1371/journal.pmed.1003207 (PMC7508401; doi:10.1371/journal.pmed.1003207)
Supplement: S1 Table — ASD, autism spectrum disorder. (DOCX) [file pmed.1003207.s004.docx]

**Table S1** Relative risk (RR) of ASD and AD in subgroups of size for gestational age (SGA: Small, AGA: Normal, LGA: Large) and sex. RR and two-sided 95% confidence intervals (CI).

| **Out**  **come** | **Size for GA** | **All - Male and female offspring** | | |
| --- | --- | --- | --- | --- |
|  |  | **22-31 weeks** | **32-36 weeks** | **≥42 weeks** |
| **ASD** | **SGA** | 2.44 (2.04-2.90) | 1.54 (1.39-1.71) | 1.41 (1.02-1.97) |
|  | **LGA** | 1.68 (1.33-2.21) | 1.34 (1.20-1.49) | 1.49 (1.05-2.11) |
|  | **AGA** | 2.36(2.18-2.56) | 1.32 (1.26-1.38) | 1.49 (1.05-2.11) |
| **AD** | **SGA** | 2.54 (2.07-3.13) | 1.35 (1.19-1.55) | 1.29 (0.86-1.92) |
|  | **LGA** | 1.40 (1.04-1.89) | 1.23 (1.08-1.40) | 1.53 (1.03-2.25) |
|  | **AGA** | 2.19 (1.99-2.42) | 1.24 (1.18-1.31) | 1.34 (1.14-1.56) |
| **Out**  **come** | **Size for GA** | **Male offspring** | | |
|  |  | **22-31 weeks** | **32-36 weeks** | **≥42 weeks** |
| **ASD** | **SGA** | 2.38 (1.94-2.91) | 1.36 (1.20-1.55) | 1.35 (0.92-1.98) |
|  | **LGA** | 1.42 (1.07-1.88) | 1.23 (1.09-1.40) | 1.49 (1.01-2.20) |
|  | **AGA** | 2.22 (2.02-2.44) | 1.25 (1.18-1.31) | 1.35 (1.16-1.58) |
| **AD** | **SGA** | 2.82 (2.14-3.72) | 1.41 (1.18-1.69) | 1.48 (0.84-2.59) |
|  | **LGA** | 1.66 (1.09-2.52) | 1.43 (1.19-1.72) | 1.74 (0.97-3.13) |
|  | **AGA** | 2.76 (2.42-3.15) | 1.38 (1.28-1.49) | 1.40 (1.09-1.79) |
| **Out**  **come** | **Size for GA** | **Female offspring** | | |
|  |  | **22-31 weeks** | **32-36 weeks** | **≥42 weeks** |
| **ASD** | **SGA** | 2.40 (1.70-3.38) | 1.87 (1.55-2.26) | 1.36 (0.72-2.60) |
|  | **LGA** | 2.28 (1.53-3.38) | 1.52 (1.26-1.85) | 1.25 (0.60-2.60) |
|  | **AGA** | 2.45 (2.09-2.88) | 1.40 (1.29-1.52) | 1.07 (0.79-1.44) |
| **AD** | **SGA** | 3.24 (2.01-5.22) | 2.49 (1.91-3.23) | 1.57 (0.59-4.16) |
|  | **LGA** | 2.52 (1.35-4.69) | 1.36 (0.97-1.90) | 1.47 (0.48-4.56) |
|  | **AGA** | 2.92 (2.29-3.74) | 1.45 (1.27-1.67) | 0.93 (0.53-1.63) |

ASD: Autism spectrum disorder, AD: Autistic disorder, GA: Gestational age; AGA: Appropriate gestational age (10^th^ to 90^th^ percentile), SGA: Small for gestational age (<10^th^ percentile), LGA: Large for gestational age (>90^th^ percentile). Relative risks (RR) estimated by log-binomial regression adjusted for country (Finland, Sweden, Norway), Birth year (1995-1999, 2000-2004, 2005-2009, and 2010-20) and Maternal age (<20, 20-24, 25-29, 30-34, 35-39, and ≥40 years)
