# Supplementary material for: Gestational age and the risk of autism spectrum disorder in Sweden, Finland, and Norway: A cohort study
Source: PLoS Med. 2020 Sep 22;17(9):e1003207. doi: 10.1371/journal.pmed.1003207 (PMC7508401; doi:10.1371/journal.pmed.1003207)
Supplement: S2 Table — ASD, autism spectrum disorder; GA, gestational age. (DOCX) [file pmed.1003207.s005.docx]

**Table S2** Relative risk (RR) of ASD and two-sided 95% confidence intervals (CI) by GA weekly versus GA week 40. Subgroups of size for gestational age and by sex.

| **Sex** | **Size for GA** | **GA week** | **RR (95% CI)** | **p-value** |
| --- | --- | --- | --- | --- |
| Female | SGA | 23 vs 40 | 7.26 (2.02-27.5) | 0.0026 |
| Female | SGA | 24 vs 40 | 9.77 (3.91-24.4) | <.001 |
| Female | SGA | 25 vs 40 | 2.41 (0.62-9.41) | 0.2066 |
| Female | SGA | 26 vs 40 | 3.20 (1.06-9.67) | 0.0395 |
| Female | SGA | 27 vs 40 | 2.97 (0.98-9.01) | 0.0552 |
| Female | SGA | 28 vs 40 | 2.48 (0.94-6.51) | 0.0657 |
| Female | SGA | 29 vs 40 | 2.41 (0.92-6.32) | 0.0749 |
| Female | SGA | 30 vs 40 | 2.19 (0.92-5.21) | 0.0764 |
| Female | SGA | 31 vs 40 | 1.87 (0.78-4.46) | 0.1590 |
| Female | SGA | 32 vs 40 | 2.54 (1.37-4.69) | 0.0030 |
| Female | SGA | 33 vs 40 | 2.56 (1.49-4.41) | <0.001 |
| Female | SGA | 34 vs 40 | 2.11 (1.31-3.40) | 0.0022 |
| Female | SGA | 35 vs 40 | 2.39 (1.66-3.43) | <.001 |
| Female | SGA | 36 vs 40 | 1.80 (1.32-2.44) | <0.001 |
| Female | SGA | 37 vs 40 | 1.90 (1.56-2.33) | <.001 |
| Female | SGA | 38 vs 40 | 1.40 (1.21-1.63) | <.001 |
| Female | SGA | 39 vs 40 | 1.11 (0.97-1.27) | 0.1247 |
| Female | SGA | 41 vs 40 | ------ | ------ |
| Female | SGA | 42 vs 40 | 1.18 (0.95-1.45) | 0.1319 |
| Female | SGA | 43 vs 40 | 1.66 (0.86-3.17) | 0.1284 |
| Female | SGA | 44 vs 40 |  | 0.9637 |
| Female | SGA | 45 vs 40 | -------- |  |
| Female | LGA | 23 vs 40 | ------ | 0.9594 |
| Female | LGA | 24 vs 40 | ------ | 0.9591 |
| Female | LGA | 25 vs 40 | 3.77 (0.97-14.6) | 0.0552 |
| Female | LGA | 26 vs 40 | 1.34 (0.19-9.34) | 0.7692 |
| Female | LGA | 27 vs 40 | 1.01 (0.14-7.06) | 0.9940 |
| Female | LGA | 28 vs 40 | 2.64 (0.87-8.05) | 0.0869 |
| Female | LGA | 29 vs 40 | 3.00 (1.14-7.86) | 0.0255 |
| Female | LGA | 30 vs 40 | 2.54 (0.96-6.68) | 0.0591 |
| Female | LGA | 31 vs 40 | 3.72 (1.96-7.08) | <.001 |
| Female | LGA | 32 vs 40 | 1.73 (0.78-3.85) | 0.1751 |
| Female | LGA | 33 vs 40 | 2.19 (1.25-3.86) | 0.0064 |
| Female | LGA | 34 vs 40 | 1.54 (0.93-2.57) | 0.0962 |
| Female | LGA | 35 vs 40 | 1.68 (1.14-2.47) | 0.0083 |
| Female | LGA | 36 vs 40 | 1.60 (1.20-2.15) | 0.0016 |
| Female | LGA | 37 vs 40 | 1.28 (1.02-1.60) | 0.0355 |
| Female | LGA | 38 vs 40 | 1.27 (1.09-1.50) | 0.0031 |
| Female | LGA | 39 vs 40 | 1.07 (0.93-1.23) | 0.3577 |
| Female | LGA | 41 vs 40 | 1.02 (0.87-1.20) | 0.8165 |
| Female | LGA | 42 vs 40 | 1.32 (1.06-1.65) | 0.0134 |
| Female | LGA | 43 vs 40 | 1.25 (0.56-2.78) | 0.5814 |
| Female | LGA | 44 vs 40 | 3.69 (0.55-24.8) | 0.1787 |
|  |  |  |  |  |

**Table S2 *Continued***

| **Sex** | **Size for GA** | **GA week** | **RR (95% CI)** | **p-value** |
| --- | --- | --- | --- | --- |
| Female | AGA | 23 vs 40 | 3.95 (1.91-8.14) | <0.001 |
| Female | AGA | 24 vs 40 | 5.38 (3.18-9.11) | <.001 |
| Female | AGA | 25 vs 40 | 4.92 (3.14-7.71) | <.001 |
| Female | AGA | 26 vs 40 | 3.51 (2.20-5.59) | <.001 |
| Female | AGA | 27 vs 40 | 2.70 (1.61-4.52) | <0.001 |
| Female | AGA | 28 vs 40 | 3.04 (1.98-4.68) | <.001 |
| Female | AGA | 29 vs 40 | 1.89 (1.14-3.11) | 0.0131 |
| Female | AGA | 30 vs 40 | 1.69 (1.06-2.71) | 0.0287 |
| Female | AGA | 31 vs 40 | 1.95 (1.33-2.85) | <0.001 |
| Female | AGA | 32 vs 40 | 2.50 (1.90-3.31) | <.001 |
| Female | AGA | 33 vs 40 | 1.37 (1.00-1.87) | 0.0512 |
| Female | AGA | 34 vs 40 | 1.48 (1.18-1.85) | <0.001 |
| Female | AGA | 35 vs 40 | 1.42 (1.20-1.69) | <.001 |
| Female | AGA | 36 vs 40 | 1.43 (1.26-1.62) | <.001 |
| Female | AGA | 37 vs 40 | 1.24 (1.13-1.35) | <.001 |
| Female | AGA | 38 vs 40 | 1.15 (1.09-1.23) | <.001 |
| Female | AGA | 39 vs 40 | 1.04 (0.99-1.10) | 0.1179 |
| Female | AGA | 41 vs 40 | 1.02 (0.96-1.08) | 0.5558 |
| Female | AGA | 42 vs 40 | 1.20 (1.10-1.30) | <.001 |
| Female | AGA | 43 vs 40 | 1.10 (0.80-1.51) | 0.5716 |
| Female | AGA | 44 vs 40 | 1.44 (0.47-4.42) | 0.5214 |
| Female |  |  |  |  |
| Male | SGA | 23 vs 40 | ------ | 0.9500 |
| Male | SGA | 24 vs 40 | 4.51 (2.14-9.53) | <.001 |
| Male | SGA | 25 vs 40 | 2.24 (0.96-5.24) | 0.0628 |
| Male | SGA | 26 vs 40 | 2.38 (1.10-5.17) | 0.0283 |
| Male | SGA | 27 vs 40 | 3.39 (1.93-5.96) | <.001 |
| Male | SGA | 28 vs 40 | 4.27 (2.69-6.77) | <.001 |
| Male | SGA | 29 vs 40 | 2.04 (1.15-3.63) | 0.0148 |
| Male | SGA | 30 vs 40 | 2.10 (1.26-3.50) | 0.0042 |
| Male | SGA | 31 vs 40 | 2.36 (1.54-3.61) | <.001 |
| Male | SGA | 32 vs 40 | 1.43 (0.88-2.32) | 0.1433 |
| Male | SGA | 33 vs 40 | 1.32 (0.88-2.00) | 0.1828 |
| Male | SGA | 34 vs 40 | 1.65 (1.23-2.21) | <0.001 |
| Male | SGA | 35 vs 40 | 1.63 (1.28-2.08) | <.001 |
| Male | SGA | 36 vs 40 | 1.39 (1.14-1.69) | 0.0011 |
| Male | SGA | 37 vs 40 | 1.38 (1.20-1.58) | <.001 |
| Male | SGA | 38 vs 40 | 1.16 (1.05-1.28) | 0.0032 |
| Male | SGA | 39 vs 40 | 1.11 (1.02-1.21) | 0.0145 |
| Male | SGA | 41 vs 40 | 1.06 (0.97-1.16) | 0.2110 |
| Male | SGA | 42 vs 40 | 1.13 (1.00-1.28) | 0.0414 |
| Male | SGA | 43 vs 40 | 1.42 (0.95-2.12) | 0.0896 |
| Male | SGA | 44 vs 40 | 3.27 (0.88-12.1) | 0.0769 |
|  |  |  |  |  |

**Table S2 *Continued***

| **Sex** | **Size for GA** | **GA week** | **RR (95% CI)** | **p-value** |
| --- | --- | --- | --- | --- |
| Male | LGA | 23 vs 40 | 3.15 (1.08-9.23) | 0.0362 |
| Male | LGA | 24 vs 40 | 0.81 (0.12-5.62) | 0.8301 |
| Male | LGA | 25 vs 40 | 1.95 (0.76-5.05) | 0.1669 |
| Male | LGA | 26 vs 40 | 1.35 (0.45-4.10) | 0.5947 |
| Male | LGA | 27 vs 40 | 0.65 (0.16-2.56) | 0.5364 |
| Male | LGA | 28 vs 40 | 1.17 (0.44-3.06) | 0.7545 |
| Male | LGA | 29 vs 40 | 1.63 (0.79-3.36) | 0.1870 |
| Male | LGA | 30 vs 40 | 2.01 (1.16-3.49) | 0.0132 |
| Male | LGA | 31 vs 40 | 1.10 (0.58-2.10) | 0.7704 |
| Male | LGA | 32 vs 40 | 1.27 (0.77-2.10) | 0.3413 |
| Male | LGA | 33 vs 40 | 0.73 (0.42-1.25) | 0.2455 |
| Male | LGA | 34 vs 40 | 0.92 (0.64-1.34) | 0.6763 |
| Male | LGA | 35 vs 40 | 1.33 (1.04-1.70) | 0.0242 |
| Male | LGA | 36 vs 40 | 1.44 (1.20-1.71) | <.0001 |
| Male | LGA | 37 vs 40 | 1.21 (1.05-1.39) | 0.0068 |
| Male | LGA | 38 vs 40 | 1.15 (1.04-1.27) | 0.0063 |
| Male | LGA | 39 vs 40 | 0.95 (0.87-1.04) | 0.2672 |
| Male | LGA | 41 vs 40 | 1.01 (0.92-1.11) | 0.8172 |
| Male | LGA | 42 vs 40 | 0.97 (0.85-1.11) | 0.6688 |
| Male | LGA | 43 vs 40 | 1.48 (0.98-2.23) | 0.0624 |
| Male | LGA | 44 vs 40 | 2.42 (0.64-9.16) | 0.1941 |
|  |  |  |  |  |
| Male | AGA | 23 vs 40 | 2.32 (1.34-4.03) | 0.0027 |
| Male | AGA | 24 vs 40 | 3.83 (2.77-5.31) | <.001 |
| Male | AGA | 25 vs 40 | 3.87 (2.96-5.07) | <.001 |
| Male | AGA | 26 vs 40 | 2.96 (2.17-4.04) | <.001 |
| Male | AGA | 27 vs 40 | 2.69 (2.04-3.54) | <.001 |
| Male | AGA | 28 vs 40 | 2.22 (1.66-2.96) | <.001 |
| Male | AGA | 29 vs 40 | 1.93 (1.46-2.56) | <.001 |
| Male | AGA | 30 vs 40 | 2.31 (1.85-2.89) | <.001 |
| Male | AGA | 31 vs 40 | 1.65 (1.31-2.08) | <.001 |
| Male | AGA | 32 vs 40 | 1.32 (1.07-1.64) | 0.0107 |
| Male | AGA | 33 vs 40 | 1.55 (1.32-1.82) | <.001 |
| Male | AGA | 34 vs 40 | 1.34 (1.17-1.53) | <.001 |
| Male | AGA | 35 vs 40 | 1.20 (1.08-1.34) | 0.0010 |
| Male | AGA | 36 vs 40 | 1.32 (1.22-1.42) | <.001 |
| Male | AGA | 37 vs 40 | 1.23 (1.16-1.30) | <.001 |
| Male | AGA | 38 vs 40 | 1.13 (1.09-1.17) | <.001 |
| Male | AGA | 39 vs 40 | 1.02 (0.99-1.06) | 0.2068 |
| Male | AGA | 41 vs 40 | 1.06 (1.02-1.09) | 0.0024 |
| Male | AGA | 42 vs 40 | 1.10 (1.05-1.15) | <0.001 |
| Male | AGA | 43 vs 40 | 1.39 (1.18-1.63) | <.001 |
| Male | AGA | 44 vs 40 | 2.42 (1.40-4.18) | 0.0016 |
|  |  |  |  |  |

GA: Gestational age; AGA: Appropriate gestational age (10^th^ to 90^th^ percentile), SGA: Small for gestational age (<10^th^ percentile), LGA: Large for gestational age (>90^th^ percentile). Relative risks (RR) estimated by log-binomial regression adjusted for country (Finland, Sweden, Norway), Birth year (1995-1999, 2000-2004, 2005-2009, and 2010-20) and Maternal age (<20, 20-24, 25-29, 30-34, 35-39, and ≥40 years), p-value: p-value associated with the test of hypothesis that week X – 40 is equal to 0 vs not equal to zero. Cells marked "------" when not estimable due to sparse data.
