# Supplementary material for: Gestational age and the risk of autism spectrum disorder in Sweden, Finland, and Norway: A cohort study
Source: PLoS Med. 2020 Sep 22;17(9):e1003207. doi: 10.1371/journal.pmed.1003207 (PMC7508401; doi:10.1371/journal.pmed.1003207)
Supplement: S3 Table — (DOCX) [file pmed.1003207.s006.docx]

**Table S3** Official birth rates by gestational age from the Finnish Institute for Health and Welfare (THL) for 2018.


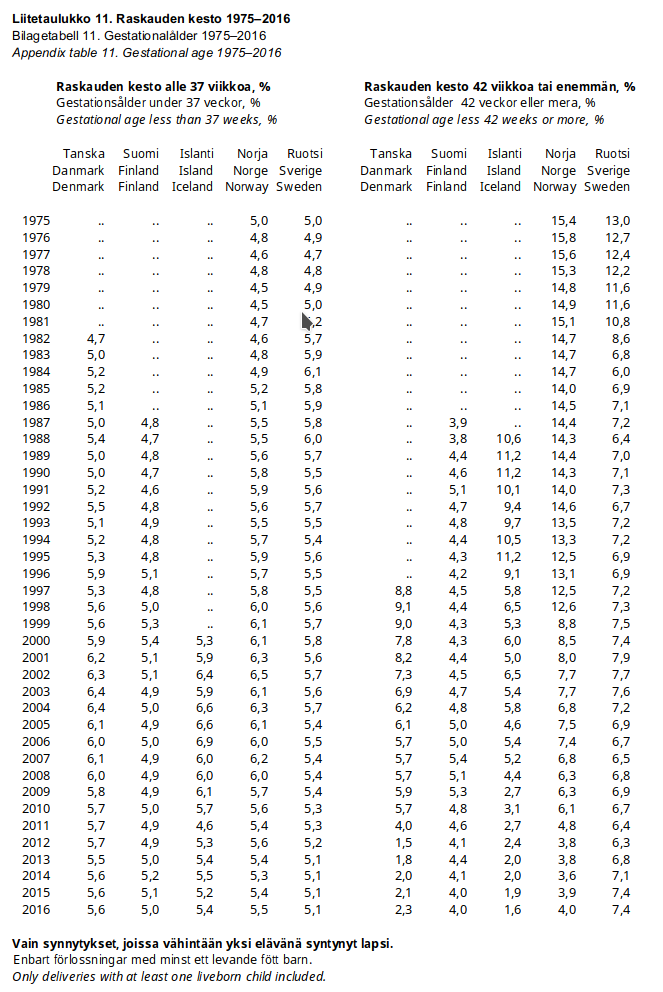


Table copied from the Finnish Institute for Health and Welfare, https://thl.fi/tilastoliite/tilastoraportit/2018/Nombir_tables_2016.pdf.
